# Emerging Applications for High K Materials in VLSI Technology

**DOI:** 10.3390/ma7042913

**Published:** 2014-04-10

**Authors:** Robert D. Clark

**Affiliations:** TEL Technology Center, America, LLC, NanoFab South 300, 255 Fuller Road, Suite 214, Albany, NY 12203, USA; E-Mail: robert.clark@us.tel.com; Tel.: +1-510-624-3478; Fax: +1-518-677-1034

**Keywords:** high K, dielectric, CVD, ALD, contacts, CMOS, DRAM, resistive RAM, diode, patterning

## Abstract

The current status of High K dielectrics in Very Large Scale Integrated circuit (VLSI) manufacturing for leading edge Dynamic Random Access Memory (DRAM) and Complementary Metal Oxide Semiconductor (CMOS) applications is summarized along with the deposition methods and general equipment types employed. Emerging applications for High K dielectrics in future CMOS are described as well for implementations in 10 nm and beyond nodes. Additional emerging applications for High K dielectrics include Resistive RAM memories, Metal-Insulator-Metal (MIM) diodes, Ferroelectric logic and memory devices, and as mask layers for patterning. Atomic Layer Deposition (ALD) is a common and proven deposition method for all of the applications discussed for use in future VLSI manufacturing.

## 1. Introduction

Over the past half century the economics of the semiconductor industry have been driven by the principle of Moore’s law, which is really the observation that as semiconductor manufacturing technology continually improves the minimum manufacturing cost per device is continually decreasing and is realized by doubling the number of devices per square unit area roughly every two years [1]. Thus, semiconductor device makers have continued to shrink or “scale” the footprint of their devices on the wafer at a relatively constant pace over the decades. The resulting increases in readily available computing power have been a boon to mankind and underpin most of the technological and scientific progress made in the past 50 years. The terminology of device nodes has arisen as a common way to reference each new two year cycle. The device node at one time equated to the half-pitch or spacing between the tightest metal lines in Dynamic Random Access Memory (DRAM) chips, then migrated to become the minimum feature size in a given chip (typically Flash memory), and now the device node is effectively a marketing term that continues to decrease linearly even if no feature on the chip can be found to match it. Nevertheless, the very real trend of doubling the number of devices per unit area biannually, first laid out by Gordon Moore in 1965, has continued steadily through nodes named in microns on to nanometer-scale nodes and very soon to nodes that one might suppose will be termed in angstroms [2].

In order to continue device scaling to the 45 nm and below nodes, semiconductor device makers have implemented High K and Metal Gate (HKMG) stacks within the Metal Oxide Semiconductor Field Effect Transistors (MOSFETs) used in digital Complementary Metal Oxide Semiconductor (CMOS) technology, which forms the basis for logic circuits within microprocessors and systems on a chip used in computers, tablets, cell phones, *etc*. [2,3]. Likewise, memory technologies, such as DRAM, have also migrated to High K dielectrics [4]. For the purpose of this review High K dielectrics refer to a class of simple binary and ternary metal oxide insulators with a relative dielectric constant greater than about 9 and comprising transition metals from groups 3–5, the lanthanides and Al. The relative dielectric constant, K, is defined according to Equation (1), where ε_d_ is the permittivity of the dielectric and ε_0_ is the permittivity of free space. Representative examples of High K dielectrics include Al_2_O_3_, HfO_2_, ZrO_2_, HfZrO_4_, TiO_2_, Sc_2_O_3_ Y_2_O_3_, La_2_O_3_, Lu_2_O_3_, Nb_2_O_5_, Ta_2_O_5_ and simple mixtures thereof. By replacing dielectrics such as SiO_2_ (K *=* 3.9) and SiON (K *=* 4–6) with High K dielectrics, CMOS and DRAM manufacturers were able to continue scaling the Equivalent Oxide Thickness (EOT) of their devices while simultaneously using a physically thicker dielectric resulting in a leakage current reduction *versus* the SiO_2_ and SiON based devices at the same EOT [2,4].

$$K=\frac{\varepsilon_{d}}{\varepsilon_{0}}$$ (1)

The use of High K dielectrics in manufacturing has paved the way for their use in applications beyond traditional logic and memory devices. As logic devices continue to evolve device makers are moving towards non-classical CMOS devices incorporating high mobility channel materials or new device architectures, which will also rely on potentially new High K dielectric stacks. Memory makers are similarly contemplating new memory devices and structures, such as Resistive Random Access Memory (ReRAM) and 3-dimensional stacked memories. In many cases the emerging applications of High K dielectrics rely on properties other than their dielectric constant. For example current conduction through the High K dielectric is used in ReRAM and controlled in many cases by ion migration within the dielectric to form conducting filaments. Because of its low deposition temperature and etch resistance, aluminum oxide may find use as a hard mask or as a sidewall spacer within double patterning schemes.

This review summarizes some of the emerging applications for High K dielectrics which may be implemented in future semiconductor manufacturing. The current status of High K dielectrics in DRAM and CMOS manufacturing is introduced as well as the commonly used deposition methods and equipment types. Future non-classical CMOS and memory devices are then described along with candidate High K dielectrics and requirements for their use. ReRAM, contact, and selector applications which make use of current conduction through the dielectrics are then introduced and discussed. The recent discovery of ferroelectric Hf/Zr oxides is discussed with potential use in logic and memory devices. Finally, the potential to use High K dielectrics, and particularly Al_2_O_3_, for patterning applications is examined.

## 2. Current Status of High K Devices

**Figure 1.**
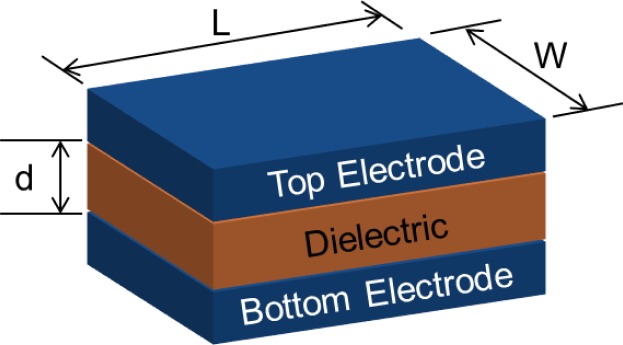
A basic parallel plate capacitor of length (*L*) and width (*W*) with spacing between the electrodes/plates (*d*).

**Figure 2.**
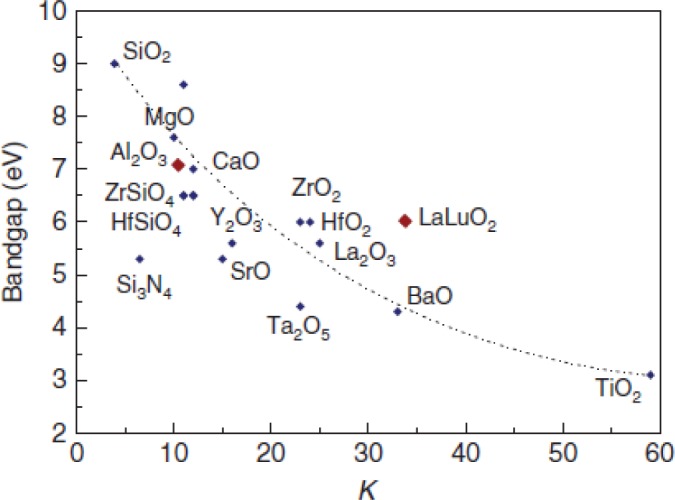
Static dielectric constant *vs*. bandgap for various High K dielectrics as well as SiO_2_ and Si_3_N_4_. Reprinted with permission from [7]. Copyright 2011 Elsevier.

As mentioned above, the main motivation for the migration to High K materials was to continue scaling the EOT of devices while maintaining a low leakage current. The conceptual advantage of a High K dielectric can best be realized by considering a simple parallel plate capacitor, Figure 1. The capacitance (C) of the device can be calculated according to Equation (2) where d is the spacing between the plates and A is the area of the device, equal to LxW. Historically the dielectric most often used in VLSI technology was SiO_2_. By substituting a High K dielectric in place of SiO_2_ the capacitance of the device can be increased for a given spacing d. In practice the High K dielectrics have a smaller band gap than SiO_2_, Figure 2, and therefore allow more current to leak between the plates unless the physical thickness of the dielectric is increased. Thus the physical thickness of the dielectric (and therefore the spacing (d)) must be increased and so a smaller reduction in EOT than what might be expected from a simple substitution of High K for SiO_2_ can be realized while maintaining the leakage current of the device [5–7]. For a given capacitance the EOT of the device can becalculated by solving Equation (2) for dwhile using the K of SiO_2_, which is 3.9, Equation (3). Thus the EOT is the thickness of SiO_2_ that would give an equivalent capacitance in accumulation to the device being measured, and is generally accepted as the “electrical thickness” of the device. In practice the situation is more complex for devices using ultra-thin dielectrics in combination with semiconductors, requiring a quantum mechanical correction to extract the accumulation capacitance, and thus extracted device parameters, such as EOT, can vary greatly depending on the methodology used [8].

$$C={K\varepsilon }_{0}\frac{A}{d}$$ (2)

$$\mathrm{EOT}=(3.9)\varepsilon_{0}\frac{A}{C}$$ (3)

### 2.1. DRAM Cell Capacitors

**Figure 3.**
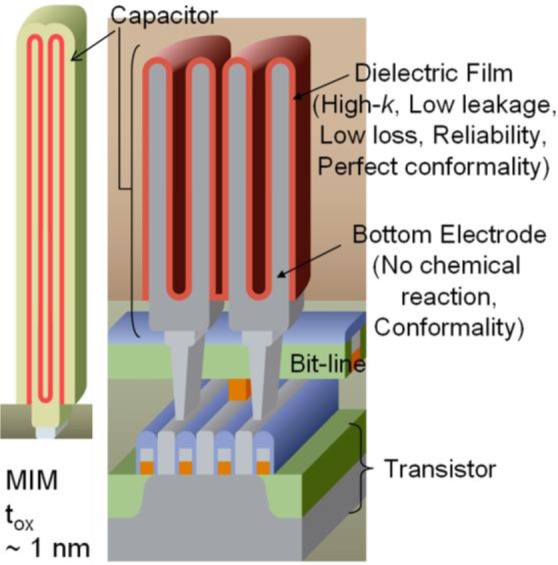
Schematic diagram of stacked Dynamic Random Access Memory (DRAM) cells with a cylindrical storage node and Metal-Insulator-Metal (MIM) capacitor stack. Reprinted with permission from [13]. Copyright 2013 WILEY-VCH Verlag GmbH & Co.

Since DRAM makes use of a capacitor as the memory element it is perhaps the most straightforward application for high K dielectrics. In DRAM the capacitor is either charged or not, corresponding to a bit value of 1 or 0 respectively. In addition it is the application that first made use of High K dielectrics in production in the 2001–2003 timeframe [4,9]. Though a modern stacked DRAM cell capacitor, Figure 3, has a much more complex shape than the parallel plate capacitor in Figure 1, it still operates in basically the same manner. The motivation for creating such a complex shape is the same as the motivation for using High K materials within the capacitor, maintaining a large cell capacitance and low leakage current while continuing to scale the footprint of the device. Referring to Equation (2), there are three basic ways to increase or maintain the capacitance of the cell as the device footprint shrinks: (1) increase the area (A) of the device; (2) decrease the dielectric thickness or spacing between the plates (d); or (3) increase the dielectric constant (K). In order to maximize the active area of the device within a shrinking footprint, the cylindrical shape shown in Figure 3 was developed. The minimum physical dielectric thickness is determined by the requirement to maintain a low enough leakage current so that the device can store a charge from one refresh cycle to the next. Though the aspect ratio of the storage nodes in DRAM capacitors continues to increase because of the shrinking footprint it is difficult to increase the active area of the device and thus the EOT of the device needs to scale aggressively without sacrificing additional current leakage. Thus, in order to reach the extremely low EOT of 3Å projected within the next few years, device makers will require higher K dielectrics with relative dielectric constants of 50 or greater [10]. Historically, DRAM manufacturers have proposed and used a variety of High K dielectric materials including Al_2_O_3_, Ta_2_O_5_, HfO_2_, and ZrO_2_, and recently have made use of a nanolaminate, termed ZAZ for its structure with a thin layer of Al_2_O_3_ sandwiched between two ZrO_2_ layers [4,9–12]. Candidates proposed for future DRAM capacitor dielectrics are generally TiO_2_-based dielectrics including perovskite type dielectrics [10] such as SrTiO_3_ and Al doped TiO_2_ [13].

### 2.2. CMOS and MOSFETs

In 2007 Intel became the first logic device maker to report Hf-based HKMG transistors in CMOS manufacturing [14]. Since then, Hf-based HKMG technology has gained wide adoption within the industry [10]. While the current status and future prospects for continued scaling of the CMOS architecture using HKMG have been recently reviewed quite extensively [2,3,7,15–20], a brief discussion of the technology is warranted here in order to introduce non-classical CMOS as an emerging application.

**Figure 4.**
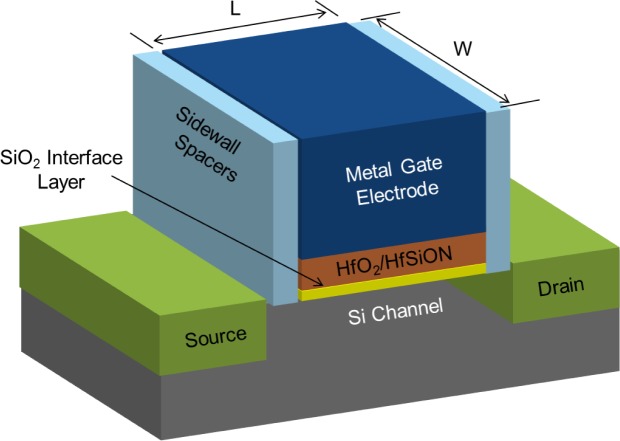
Schematic diagram of a basic planar High K and Metal Gate (HKMG) Metal Oxide Semiconductor Field Effect Transistors (MOSFET) showing common Hf-based gate stacks with a SiO_2_ Interface Layer. The Gate Length (*L*) and Channel Width (*W*) are labeled at the top of the Metal Gate Electrode.

A basic planar bulk HKMG transistor, illustrated graphically in Figure 4, consists of a Si Channel bounded by the Source and Drain, and insulated from the Metal Gate Electrode by a Gate Dielectric comprised of a very thin SiO_2_ interface layer and a Hf-based High K layer. The thin SiO_2_ layer under the High K is required in order to maintain the reliability of the transistor and in order to maintain the carrier mobility in the channel, see below. Within the bounds of the Sidewall Spacers the HKMG stack forms a parallel plate capacitor with the Metal Gate Electrode and Si Channel as the top and bottom electrodes respectively. Since the gate dielectric is a nano-laminate of SiO_2_ and High K dielectric it has an effective K somewhere between the two and can be estimated by a linear combination based on physical thicknesses if needed. It is worth noting that the typical physical thicknesses of the interface (~6–10 Å) and High K (~15–20 Å) layers in current state of the art MOSFETs are pushing the limits of what can be measured accurately even with state of the art metrology, so electrical characterization is generally relied upon more heavily. From an electrical perspective the interface layer and high K layer can be treated as two capacitors connected in series and thus the total dielectric/oxide capacitance C_ox_ can be found from Equation (4) where C_IL_ and C_HiK_ are the capacitances of the Interface and High K layers respectively. Combining with Equation (3) leads to a very useful approximation, Equation (5), that the total EOT of the device is equal to the EOT of the Interface Layer plus the EOT of the High K Dielectric.

$$\frac{1}{C_{\text{ox}}}=\frac{1}{C_{\text{IL}}}+\frac{1}{C_{\text{HiK}}}$$ (4)

$$\mathrm{EOT}={\mathrm{EOT}}_{\text{IL}}+{\mathrm{EOT}}_{\text{HiK}}$$ (5)

$$I_{\text{ds}}=\frac{1}{2}{\mathrm{\mu C}}_{\text{ox}}\frac{W}{L}{\left(V_{g}-V_{t}\right)}^{2}$$ (6)

A MOSFET works as a solid state switch by applying a voltage across the source and drain. When no charge is applied to the gate electrode, no current flows to the drain from the source. As a voltage is applied to the gate electrode carriers are attracted to the surface of the Si channel and current can flow from the source to the drain. For a long channel device the current flow will saturate to a value *I*_ds_ according to Equation (6) where μ is channel mobility or how fast charge carriers can flow through the channel, *V*_g_ is the gate voltage relative to the source, and *V*_t_ is the threshold voltage which is nominally the voltage at which the device switches from off to on. It is worth pointing out again that the relations above are simplistic and based on idealized devices, and do not apply directly to devices scaled to the dimensions currently used, but they are useful in understanding the factors influencing device performance and a good deal of device engineering effort is expended yearly trying to maximize the transistors drive current (Ion) while maintaining an acceptable off current (Ioff).

**Figure 5.**
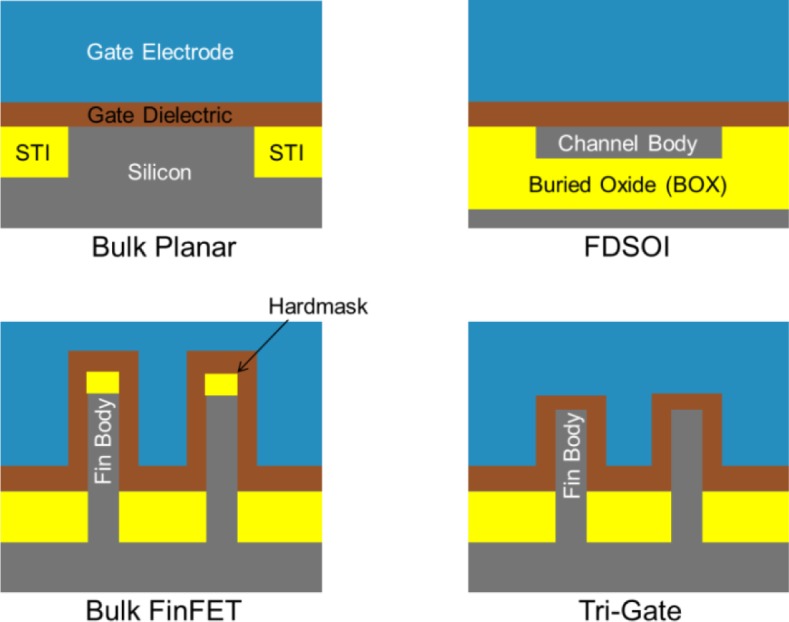
Schematic cross-sections across the channel, looking from source to drain, of the transistor comparing traditional Bulk Planar with Fully Depleted Silicon on Insulator (FDSOI), Bulk FinFET and Tri-Gate device architectures which have been or will be implemented at the 22 nm and below device nodes.

To form a CMOS inverter, the basic building block of CMOS logic, two different “flavors” of transistor are required to be connected together, an N-type MOSFET (NFET) and a P-type MOSFET (PFET), distinguished from each other by the polarity of the charge carriers which are electrons (negative) and holes (positive) respectively. In order to continue scaling the planar MOSFET without deleterious short channel effects it has traditionally been necessary to continue scaling the electrical thickness or EOT of the device along with the physical dimensions of the device according to a general relation first proposed by Robert Dennard and his colleagues at IBM in 1974 [21]. Dennard’s scaling rules were followed for decades on MOSFETs with SiO_2_ gate dielectrics to simultaneously reduce the size of the transistor and improve the switching speed and delay of the device as it became smaller resulting in chips that ran at ever faster clock rates. However, at the 90 nm node, the SiO_2_ gate dielectric had scaled to a thickness of just 1.2 nm, equal to only about four molecular layers of SiO_2_and the power dissipation and heat of the chips, which had originally been effectively constant, had begun to rise alarmingly due to leakage currents and resistance. At that point the leakage through the gate dielectric became too high to continue scaling its physical thickness, so at 65 nm the gate dielectric failed to scale, and it became necessary to introduce High K dielectrics at the 45 nm and below nodes as mentioned above. However, even with High K dielectrics it has not been possible to continue scaling planar bulk MOSFETs below the 20 nm node for leading edge device makers, primarily because the EOT of the gate dielectric cannot be scaled according to Dennard’s scaling rules. In fact, the era of improving transistor performance according to Dennard scaling has passed and device makers are now using new knobs beyond pure dimensional scaling to improve device performance. In order to make up for the lag in EOT scaling device makers have introduced strained Si technology at 90 nm and below nodes which improves the mobility of the transistor by straining the Si channel, and at the 22 nm node and below device makers are introducing fully depleted device architectures that have improved short channel effects enough to allow the channel length to scale without scaling the dielectric EOT, Figure 5.

Referring to Figure 5 the new device architectures being implemented at 22 nm and beyond are all fully depleted device architectures because the Si Body thickness is less than the depletion length of Si majority charge carriers in each case [22–25]. The benefit of fully depleted architectures is an effective lowering of the *V*_t_ of the device *versus* a bulk planar device and simultaneous reduction in the Ioff due to lower leakage between the source and drain. Typically CMOS operates using a supply voltage (*V*_dd_) of about three times the *V*_t_, so lowering the *V*_t_ allows a lower operating voltage, resulting in significant power savings for the same performance or significantly higher performance at the same power level. While all of these devices outperform Bulk Planar MOSFETs with similar dimensions there are some differences in how they operate, and the performance that can be expected from each, Table 1. The Fully Depleted Silicon on Insulator (FDSOI) architecture [24] is the most similar to Bulk Planar and therefore seems to offer the least additional process complexity. The double gated Bulk FinFET [25] is distinguished from the triple gated Tri-Gate [22,23] by being relatively taller and thinner, and having a hardmask left in place over the fin, meaning the gate only acts on the sides of the fin *versus* the sides and top as in the Tri-Gate.

**Figure 6.**
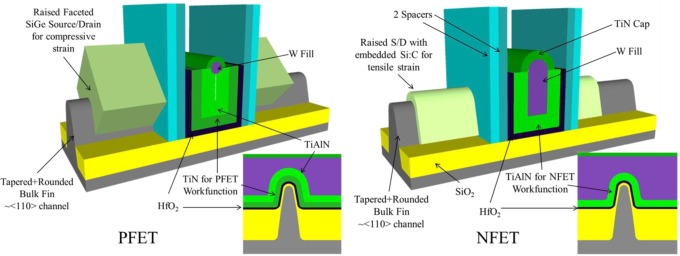
Schematic diagrams of Intel 22 nm HKMG Tri-Gate P-type MOSFET (PFET) and N-type MOSFET (NFET) showing major performance elements and insets of schematic cross-sections showing gate stack detail for each device. Based on [22,23,26,27].

In practice, Intel’s Tri-Gate transistor, which is the only fully depleted transistor technology in high volume manufacturing at the time of this writing, is tapered and rounded at the top in such a way that there is no flat top gate, Figure 6. Generally the bulk FinFET and Tri-Gate devices are quite similar in terms of their operation and offer better electrostatic control, as evidenced by the lower Drain Induced Barrier Lowering (DIBL) and Subthreshold Swing (SS), than FDSOI. However, the FDSOI device has a unique advantage not represented in Table 1 in that this architecture allows designers to put a charge on the Si substrate underneath the thin Buried Oxide (BOX), termed Back Biasing, to dynamically raise or lower the performance (and power) of a block of logic which is quite attractive for certain applications. The performance advantage of FinFET and Tri-Gate, as evidenced by higher Ion *versus* Device Footprint, is primarily due to the 3-dimensional structure of the devices. The effective channel width (Weff) of a fin, as used in Equation (6) and referring to Figure 5, would be twice the fin height, and for a Tri-Gate twice the fin height plus the fin width. Therefore, the effective device channel width (Weff) is actually larger than the device footprint width, resulting in a higher drive current *versus* device footprint. In a real device the structure is not quite so simple, Figure 6, since the shape of the fin is not so regular, but still results in a higher drive current per device footprint even though the Tri-Gate and FDSOI devices have quite a similar drive current per Weff, Table 1. The fin pitch also plays a large role because Weff is effectively quantized for FinFET and TriGate devices- the device can have three or four fins, but not 3.5. The process complexity for manufacturing these devices should be evident when considering structures of the NFET and PFET depicted in Figure 6 and the device dimensions, Table 1. The performance requirements for the Hf-based High K dielectric layer include near perfect conformality and continuity along with minimal thickness variation across a 300 mm wafer, typically <1% Within Wafer Nonuniformity (WIWNU) at the 1σ level is allowed for High K gate dielectrics within the industry for a film that is <2 nm thick.

## 3. Deposition

There are three deposition methods for High K dielectric layers that have been employed for VLSI manufacturing: Physical Vapor Deposition (PVD), Chemical Vapor Deposition (CVD) and Atomic Layer Deposition (ALD). For High K dielectric deposition, CVD and ALD, which are conceptually-related to one another, make up the bulk of the market and use similar equipment, while PVD has seen relatively little use in manufacturing despite extensive use in research and development. Each of these methods will be discussed briefly here and has been treated in more detail elsewhere specifically with respect to Hf silicate and HfO_2_ used in CMOS manufacturing as well as HfO_2_, ZrO_2_ and Al_2_O_3_ used for DRAM manufacturing [12,16,18,28–33].

### 3.1. Physical Vapor Deposition (PVD)

PVD, or sputtering, is a high energy process where a target of the material or alternately the base metal of interest is bombarded with an inert plasma in order to evaporate it onto the wafer. PVD generally requires very high vacuums on the order of 10^−7^ Torr or less due to the low vapor pressure of the evaporated materials, and the substrate is generally maintained at room temperature during the deposition. PVD of high K oxides may employ the oxide with a completely inert atmosphere or may be so-called reactive sputtering, which employs the base metal with some oxygen in the atmosphere in order to form the oxide of interest. For instance La_2_O_3_ could be sputtered from a starting target of La_2_O_3_ or from a starting La metal target if oxygen were included in the atmosphere of the chamber in order to react the La metal during deposition. In some cases it has been found that even with an oxide target some oxygen may be required during the deposition in order to preclude silicide formation during deposition [34]. Because PVD is a line of sight technology, it is not well suited to coating high aspect ratio 3-Dimensional structures. In addition, the high ion bombardment during PVD processes is thought to damage the Si Channel in MOSFET devices if it is used to deposit the main High K layer resulting in mobility degradation. However, PVD has found use for deposition of the *V*_t_ adjusting cap layers used in gate first planar MOSFET devices [7,35–39]. In this application the cap layer is deposited on top of the HfO_2_ High K gate dielectric, which serves to protect the Si channel. The intricacies of gate first *versus* gate last integration are beyond the scope of this paper, but the high thermal budget of the gate first integration scheme is required for the cap layer to diffuse through the HfO_2_ where it alters the dipole at the High K/SiO_2_ interface resulting in a band edge work function for the NFET device [7,36,37,39–47]. Two trends in CMOS manufacturing are pointing toward the fading of the use of this method in the future: (1) Device makers are becoming more likely to use the lower thermal budget gate last integration scheme which will not allow the cap layer to diffuse and alter the *V*_t_; and (2) Device makers are migrating to 3-dimensional transistor structures such as FinFET that are more difficult to coat conformally using PVD. If PVD can no longer be used due to the high aspect ratio of the CMOS structure, it has already been shown that these layers can be deposited by ALD, with the added benefit of enabling *V*_t_ layers to be inserted within the HfO_2_ gate dielectric for lower thermal budget [47–49]. Of course PVD does have some advantages over CVD and ALD in terms of its flexibility, low cost and low temperature. Nearly any High K material imaginable, up to complex quaternary oxides can be deposited easily by PVD at low temperature in a research environment. This flexibility has enabled countless materials screening studies for early pathfinding in the industry. Thus, regardless of whether it is used for High K dielectric deposition in future manufacturing nodes, PVD will continue to be used for R&D purposes.

### 3.2. Chemical Vapor Deposition (CVD) and Atomic Layer Deposition (ALD)

**Figure 7.**
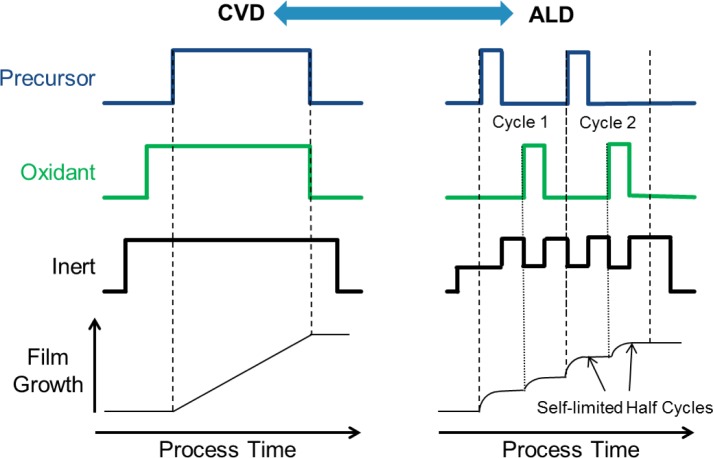
Process Schematic showing a basic gas flow sequence to the chamber for Chemical Vapor Deposition (CVD) and for Atomic Layer Deposition (ALD) as well as expected film growth profiles *vs*. process time. For illustration purposes, two ALD cycles are shown and labeled, including half cycles, but the cycles may be repeated indefinitely in practice to obtain the desired film thickness.

CVD and ALD are very closely related to one another conceptually, and in some cases they exist simultaneously, though that is usually considered detrimental in the case of ALD. In fact, ALD can reasonably be considered as a variant of CVD [18,30]. CVD and ALD for High K dielectrics use similar hardware, operate in similar pressure and temperature ranges and use similar precursors. Typical deposition processes, with respect to gas flows to the deposition chamber, are shown in Figure 7. As can be seen the processes are distinguished from one another by whether or not the precursor and oxidant are present in the chamber at the same time. For the purpose of this review, the term oxidant, or oxidizing agent is the chemical agent providing the oxygen during the deposition process, and is not intended to imply a formal reduction-oxidation chemical mechanism. In ALD the precursor and oxidant enter the deposition chamber separately and are never present together in the gas phase due to inert gas purges between the precursor and oxidant pulses. In CVD the precursor and oxidant are both present in the chamber during the deposition. While plasma enhanced versions of both CVD and ALD are known for High K dielectrics, in production thermal CVD and ALD have been preferred traditionally, and used in VLSI manufacturing. Another distinction between CVD and ALD is the nature of the deposition. While CVD typically produces a continuous deposition rate with respect to time, ALD is a cyclical deposition method wherein each half cycle is self-limited due to the surface chemistry such that extending the time of the precursor or oxidant pulse beyond a saturated pulse does not result in significant additional film growth. Typical ALD processes for High K dielectrics produce something on the order of a third of a monolayer or less per cycle (on the order of about 1 Å per cycle or less) [31,48].

CVD has found limited use for High K dielectric deposition in manufacturing. The high aspect ratios of DRAM structures are not amenable to CVD depositions, and the ALD method is preferred for those structures. For High K gate dielectrics CVD has found a role in the deposition of Hf silicate films, which are typically then nitrided to form HfSiON [50]. While the dielectric constant of HfSiON is not quite as high as pure HfO_2_, HfSiON is more similar to the traditional SiON, is more thermally stable in contact with Si, and has a larger band gap than HfO_2_. However, much like for PVD, the trends in VLSI manufacturing are pushing away from CVD and toward the use of ALD for High K gate dielectrics due to the higher aspect ratio features inherent in FinFET and Tri-Gate devices, the lower thermal budgets used for gate last integration, and the desire to scale the EOT by employing the Higher K value of pure HfO_2_ in future devices. Thus, ALD is expected to dominate High K dielectric deposition in manufacturing for future VLSI applications.

The self-limited nature of ALD provides significant advantages for semiconductor manufacturers. Because the film growth is digital the film thickness can be set by selecting the number of deposition cycles. The deposition rates, particularly for Hf, Zr and Al oxides as used in production, are determined by the surface chemistry, and small variations in temperature, pressure or pulse time (provided a margin is maintained ensuring a saturated pulse) have relatively little effect on the growth per cycle of the process within the ALD process window, especially when compared with CVD depositions. Thus, wafer to wafer, lot to lot, and tool to tool variations are at least theoretically easier to control for ALD processes. In addition, near perfect conformality can be realized even in high aspect ratio structures—suitable for any of the three dimensional structures discussed herein. Because the precursor and oxidant are separated, it is possible to use more reactive precursors and oxidants in the ALD process as well since the reactant separation minimizes the possibility of gas phase reactions that can lead to particle formation in the case of CVD. Finally, ALD tends to operate in a slightly lower temperature regime than the corresponding CVD process and so it can provide a lower thermal budget as well. For the emerging applications discussed below, some combination of each of these advantages suggests that ALD is likely to be employed if and when these new devices enter manufacturing.

### 3.3. Deposition Equipment Styles

**Figure 8.**
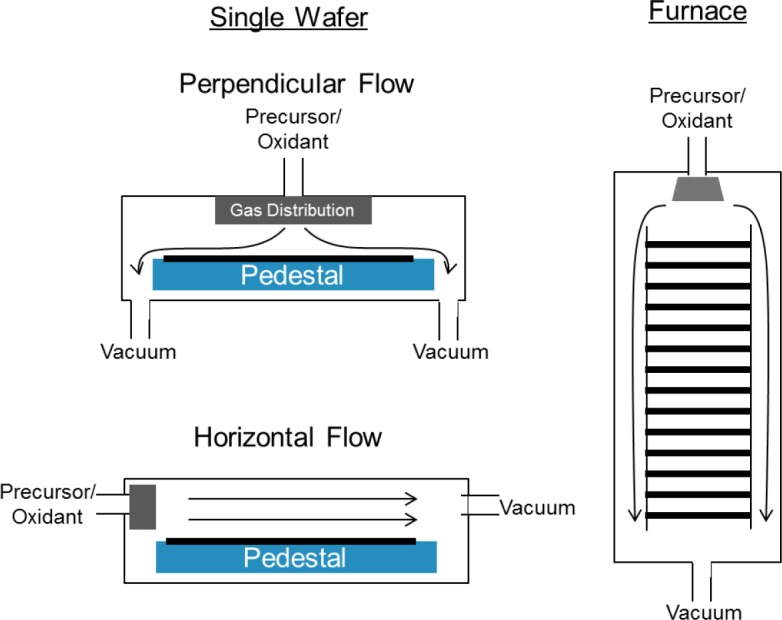
Schematic diagram illustrating basic equipment styles used in VLSI manufacturing for CVD and ALD of High K dielectrics.

Deposition equipment used for CVD and ALD of High K dielectrics can be categorized within a few basic configurations, Figure 8. For DRAM dielectrics device makers tend to prefer furnace, or batch style, systems because they are more cost effective for the thicker dielectric layers used in DRAM, and because the product itself is more cost sensitive. Single wafer systems tend to be used for gate dielectric depositions which require the best uniformity, are typically thinner, and are less cost sensitive. In single wafer configurations the chamber may be configured for cross-flow or perpendicular flow. Both types have been used for ALD, but for CVD the perpendicular flow style is more common, and typically the gas distribution is through a showerhead. For ALD systems gas distribution is less critical and can be accomplished with a simple cone to allow the gas to expand or with a small showerhead. Equipment design considerations can vary somewhat depending on the particular precursor and process.

## 4. Emerging Applications

Multiple new applications are currently contemplated for High K dielectrics. Within CMOS and DRAM manufacturing there is a push to develop Higher K gate dielectrics in order to reduce EOT and increase capacitance in the devices. In addition, future CMOS devices may make use of alternative channel materials, which will require new gate dielectric stacks in order to meet EOT and device performance targets. Beyond CMOS the possibility for using Tunnel FETs and other steep sub-threshold slope switches is under investigation. Below the 14nm nodes contact resistance within CMOS is becoming a larger issue, and High K layers have been proposed for Metal-Insulator-Semiconductor (MIS) contact schemes that alleviate the Fermi level pinning phenomenon. New memory devices, in the form of Resistive RAM (ReRAM) and Ferroelectric FETs incorporating traditional High K dielectrics are proposed for future nonvolatile memories as well. MIM diodes have also recently been investigated which may find use as selection devices in ReRAM or other future nonvolatile memory candidates. Building on the foundation of High K dielectrics in VLSI manufacturing discussed above, each of these areas is introduced below briefly.

### 4.1. Higher K Dielectrics and High Mobility Channels in CMOS

Referring to Equation (5) above, reducing the EOT of the gate dielectric stack can be accomplished in several ways, but must result in a net reduction in the EOT of the interface layer or the High K layer, or some combination thereof. The potential for using Higher K dielectrics, as well as Higher K and scaled interface layers was recently reviewed extensively in this journal [15] as well as elsewhere [7,18]. Most dielectrics with a higher K than HfO_2_ result in an unusable Effective Work Function (EWF), meaning the *V*_t_ for the PFET and NFET cannot be set near enough to the midgap of Si to allow the CMOS architecture to function. Therefore, optimizing the interface layer thickness for EOT minimization, while maintaining EWF control, mobility and reliability, has become the main focus for EOT scaling in Si based devices.

One potential alternative High K dielectric which does not suffer from the problem with the EWF shift has recently seen renewed interest as well, namely ZrO_2_. ZrO_2_ is infinitely miscible with HfO_2_, and due to their well-known similarity, Zr and Hf tend to have analogous precursors that do not react with each other deleteriously during ALD. Thus it is possible to form mixed Hf-Zr oxides easily with any desirable ratio of Hf:Zr by ALD [48,51–53]. It has further been found that, while HfO_2_ typically crystallizes in the lower K monoclinic form, ZrO_2_ tends to crystallize in the tetragonal form which is thought to have a higher K [54,55]. Thus, doping ZrO_2_ into HfO_2_, or using pure ZrO_2_ is one potential way to boost the dielectric constant of the High K dielectric stack. In addition gate stacks incorporating ZrO_2_ along with HfO_2_ exhibit improved reliability, mobility, and charge trapping [56–59]. Thermal budget control is critical in using ZrO_2_ in the gate stack, as ZrO_2_ tends to be more thermally reactive than HfO_2_ [60–62]. But, as mentioned above, the current trend towards gate last engineering brings with it a lower thermal budget for the HKMG stack.

Another approach to future CMOS is to improve electrostatics even further by employing a Gate-All-Around FET (GAA-FET) structure [3,17,19,20,63–69]. Such a structure should allow the extension of the Si channel to beyond the 10 nm node, while continuing to employ the traditional High K gate dielectrics in use today, and therefore can be considered the most likely scenario for scaling beyond the 10 nm node. This structure uses nano-wire Si as the MOSFET channel and requires the gate dielectric and metal gate to wrap completely around the nanowire. Using ALD for the gate dielectric and work function metals, such a structure is thought to be manufacturable. Eventually though, the need for scaling EOT will present itself again, or the transistors drive current will need to be increased by another means.

Beyond the use of Higher K gate stacks and GAA-FETs another potential increase in transistor performance may come from substituting higher carrier mobility semiconductors for Si within the MOSFET channel [3,19,20,70]. The leading candidates for high mobility channel materials include Ge for the PFET channel [71,72] and III–V materials, particularly InGaAs for the NFET channel of the MOSFET [73,74]. Again, ALD is well suited to meet the challenges associated with forming gate stacks on high mobility substrates [75,76].

Ge PFETs have shown particular promise, and as Ge is a fab friendly group 14 material, like Si, and is already used in mainstream manufacturing, integrating Ge PFETs along with Si NFETs seem to be apromising path for the first implementation of high mobility channel materials in manufacturing. In fact, SiGe channel PFETs are already being used in current generation CMOS; SiGe is arguably a high mobility channel material as well, and represents an incremental step towards using a pure Ge channel [24,77]. In the case of both pure Ge and SiGe, HfO_2_ is well established as the leading candidate for the bulk of the High K dielectric. The use of a Si cap over the Ge channel seems to represent a leading approach for passivating interface defects and allows the use of the same High K dielectric stacks used for Si [71,72]. GeO_2_ has also been proposed as a passivation and interface layer for High K/Ge gate stacks [78–85]. This approach is attractive due to its apparent similarity to the Si/SiO_2_ system traditionally used; however process control of the Ge oxidation is complicated by the delicate nature of the oxide. Another approach is to employ an Al oxide interface layer for Ge surface channels [86]. One attractive aspect of this approach is the ability to scale the EOT due to elimination of the SiO_2_(or GeO_2_) interface layer and substitution for a higher K interface. Another attractive aspect of an Al_2_O_3_ interface layer on Ge, is the potential to use the same interface layer material with a III-V NFET, as described below. Ge also has some interesting benefits with ZrO_2_ as the gate dielectric [87–89]. As with the III–V material systems discussed below the passivation of defects at the Ge/High K interface is a key challenge for any High K dielectric scheme used, which can be largely avoided by using a Si cap [90–94]. However, use of a Si cap for passivation has some detriments as well. If GAA-FETs are employed, it will be necessary to develop conformal Si capping processes presumably either using ALD or atomic layer epitaxy. Such processes, to this author’s knowledge, have not been well established. In addition the conduction band alignment of Si with Ge is such that electrons would not be confined in the Ge channel, meaning this approach is not useful if Ge NFETs are desired. However, if Ge PFETs are implemented prior to GAA-FETs then it is likely to be accomplished using Si passivation layers over a Tri-Gate or FinFET type device for a PFET in combination with a strained Si NFET.

As mentioned above Ge NFETs are a possibility for future CMOS manufacturing as well, and matched with Ge PFETs would represent perhaps the most feasible single channel high mobility CMOS candidate [80,81,95–101]. Provided that a lower *V*_dd_ can be implemented in order to maintain a low Ioff, due to the lower band gap of Ge, two major hurdles exist to this technology: (1) Metal/Ge contacts tend to pin at the valence band edge which is ideal for contacts to Ge PFETs, but results in unusably high resistance in Ge NFET contacts; and (2) The defects near the conduction band edge need to be effectively passivated without a Si passivation layer, requiring development of High K gate stack processes that can accomplish this goal [100]. One possible approach to overcoming the high Ge NFET contact resistance problem is the use of an MIS contact structure incorporating a High K dielectric, discussed below. If single channel Ge CMOS with acceptable performance can be demonstrated with a common gate stack for NFET and PFET, then it would become an attractive option for future VLSI manufacturing due to its cost savings *versus* implementing dual channel CMOS. In addition, incorporating Sn into the Ge channel has been proposed for extending Ge CMOS to future generations with even higher mobility channels [102].

The possibility for III–V NFETs due to their high electron mobility has also been extensively investigated and reviewed [18,70,92,94,103–105]. A benchmark result reported by Intel in 2011 employed a quantum well structure within a Tri-Gate architecture and a TaSiO_4_ gate dielectric [73]. This important result demonstrated that reasonable electrostatic control and performance was possible in III-V NFETs. It still remains to be seen if scaled III–V NFETs can be fabricated that have performance exceeding the performance possible with highly scaled strained Si NFETs. However, several very recent results are worth highlighting, as they have exceeded the record performance first reported by Intel. Groups at MIT, TSMC, and UC Santa Barbara along with their collaborators have recently reported record transconductance (Gm) of about 2.7 mS/μm, which exceeds the highest Gm in III-V MOSFETs reported to date [106–111]. SEMATECH also presented a benchmark result recently with excellent short channel effects (SS = 77 mV/dec. and DIBL = 10 mV/V) for short channel devices with Gm greater than 1.5 mS/μm in a quantum well Tri-Gate MOSFET which represents the best Gm in a III-V MOSFET attained with a SS below 80 mV/dec in a potentially manufacturable device [112]. The MIT, SEMATECH and UC Santa Barbara groups all make use of ALD HfO_2_ as the bulk high K dielectric. While the High K dielectric used by TSMC was not reported, it was deposited by ALD. SEMATECH and UC Santa Barbara have employed Al_2_O_3_ as the interface and passivation layer, while the MIT group employed InP underneath the HfO_2_ for passivation. These results strongly suggest that future III–V devices used in manufacturing would continue to employ HfO_2_ as the gate dielectric, and that a dual channel solution with a Ge PMOSFET and a common High K Gate Dielectric stack incorporating Al_2_O_3_ and HfO_2_ might be possible.

The possibility of using III-V semiconductor channels in PFETs hinges on Sb based systems including GaSb, InSb, and InGaSbdue to their high hole mobilities [113,114],and III-Sb channels have been proposed as a potential single channel solution for CMOS [115]. However, these systems are not as well developed for NFETs and the III-As systems, nor for PFETs as the Ge channel devices, so it is not considered a near term solution for CMOS manufacturing.

For solutions beyond the III–V and Ge channel systems 2-Dimensional channel materials as well as carbon nanotubes have garnered interest. These “beyond the roadmap” materials will all require High K gate dielectrics that can be used without compromising the channel integrity. Related C-based systems including graphene and nanotubes have been investigated [116–123]. In addition, there are many non-C analogs to graphene that have been proposed as potential channel materials, particulary the metal dichalcogenides [124–128]. Device makers are also contemplating what might replace the CMOS architecture, and how to make switches that can give steeper subthreshold slopes than traditional MOSFETs, with Tunnel FETs frequently mentioned as a possibility due to their similarity to traditional MOSFETs and extremely low SS [3,19]. Any of these materials and devices will take at least a decade to make its way to becoming a leading candidate for CMOS insertion/replacement on the ITRS roadmap, and certainly other potential solutions exist currently and will be found along the way. The important point made by this type of research is that there are potential logic solutions on the horizon for years to come.

### 4.2. MIS Contacts

**Figure 9.**
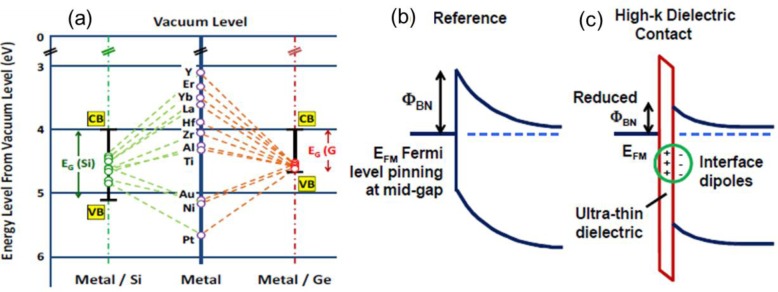
(**a**) Most metal-semiconductor contacts result in Fermi level pinning to mid-gap on Si and the valence band edge on Ge; (**b**) fermi level pinning at mid-gap results in a large Schottky Barrier Height (SBH) adding resistance; (**c**) inserting a dielectric layer at the interface reduces MIGS penetration resulting in less Fermi level pinning and SBH can be further tuned by interface dipoles. Reprinted with permission from [132]. Copyright 2012 IEEE.

As mentioned above contact resistance is becoming increasingly problematic for the 14 nm and below geometries [10]. The problem arises from a confluence of factors including Fermi level pinning at the metal-semiconductor interface, difficulty in doping high aspect ratio and ultra-thin body structures, fundamental limits on doping due to solid solubility limits, and contact area reduction due to scaling or new device structures. MIS Contacts, where an ultra-thin (typically < 1 nm) High K dielectric layer is inserted between the metal and semiconductor to form an MIS structure, have been proposed as a potential solution to this problem [129–131]. In this case, the lower band gap High K dielectrics are preferred as the objective is for the dielectric to contribute as little resistance to the contact structure as possible. The idea of inserting a dielectric into a metal-semiconductor contact in order to reduce resistivity is counter-intuitive, and so the operating principle deserves some explanation. Referring to Figure 9, inserting a thin dielectric layer between a metal-semiconductor contact can de-pin the interface and lower Schottky Barrier Height (SBH) by limiting Metal Induced Gap States (MIGS) penetration. Interface dipoles can further reduce the SBH in some cases, but the main effect, especially with high doping depends mostly on reducing MIGS penetration [132–137]. The leading candidate applications for this technology in the near term are N-type contacts to Si and Ge, for which TiO_2_ has been found to be a particularly good candidate [136–141]. In addition this technology has been proposed for metal/III–V contacts including III-As and III-Sb systems [142,143]. Contributing factors to the implementation of this technology could be the increasing difficulty of forming NiSi within high aspect ratio structures as well as the decreasing benefit of NiSi due to lower contact area enhancement on Fin-type devices.

### 4.3. Resistive RAM (ReRAM)

**Figure 10.**
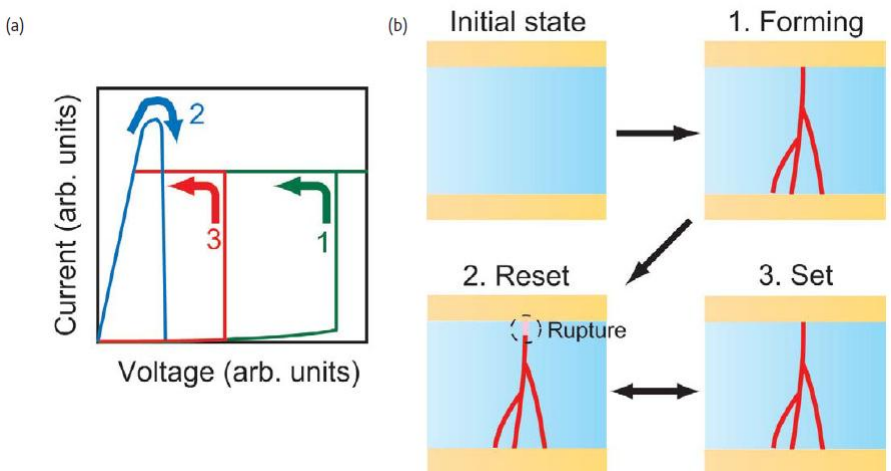
(**a**) Current-Voltage behavior for a unipolar ReRAM during (1) Forming; (2) Reset and (3) Set processes. After forming the device is cycled between Set and Reset as it is written. (**b**) Schematic of physical processes during (1) Forming; (2) Reset and (3) Set processes. Oxygen vacancies migrate under the applied voltage during Forming and Set processes to form the conducting filament, and the filament is broken by resistive heating from high current flow during Reset. Reprinted with permission from [144]. Copyright 2008 Elsevier.

Resistive RAM (ReRAM) is an emerging non-volatile memory technology that potentially employs traditional high K dielectrics [12,144–146]. The structure of a ReRAM memory stack is basically the same as an MIM capacitor, with a High K dielectric sandwiched between two metal electrodes. For traditional High K dielectrics in ReRAM structures, such as ZrO_2_, HfO_2_, Ta_2_O_5_ and TiO_2_, the resistive switching mechanism is thought to be filamentary. Referring to Figure 10, for a unipolar filamentary ReRAM, during the forming process a conducting filament is initially formed under high voltage in a process related to oxygen vacancy migration in the dielectric layer. Controlling the compliance current is useful for controlling the on state or low resistance state current level. After forming, flowing a high current through the ReRAM device causes joule heating allowing oxygen vacancies to redistribute and rupturing the filament. The filament can be re-formed by using a set voltage, again with a lower compliance current than the reset process.

Multi-level cross point arrays have been proposed for implementing ReRAM memories, with a CMOS front end that can be used for controlling and selecting the various memory block formed [147–149]. Since the cross point arrays can be formed within back end metal line and via structures and stacked on top of one another, a high density non-volatile memory is the result. Tight electrical parameter control and effective design of the dielectric stack to control oxygen vacancy movement in order to meet endurance and repeatability criteria are required in order for these devices to be implemented in manufacturing [150,151]. Multi-layer High K stacks including an O vacancy well, nearly stoichiometric HfO_2_ as the O vacancy medium in which the filament forms and an O vacancy deficient layer, such as Al_2_O_3_ to control filament rupture have recently been proposed to meet these requirements [152]. The O vacancy well can be formed by reaction of HfO_2_ with a highly electropositive metal such as Hf or Ti, sometimes called an oxygen exchange layer (OEL). Depositing the High K dielectric layers by ALD into the high aspect ratio structures proposed is considered relatively routine based on the current state of the art described above, but the OEL is more difficult by ALD. However, there is at least one recent report of forming a working ReRAM device using HfO_2_ and a Ti metal OEL both deposited by ALD [153]. Thus, the potential for manufacturing high density ReRAM using highly scaled 3-dimensional crosspoint structures has been shown in principle, though realizing a stack by this method that has the reliability needed for a commercial product has not yet been demonstrated.

### 4.4. MIM Diodes as Select Devices?

**Figure 11.**
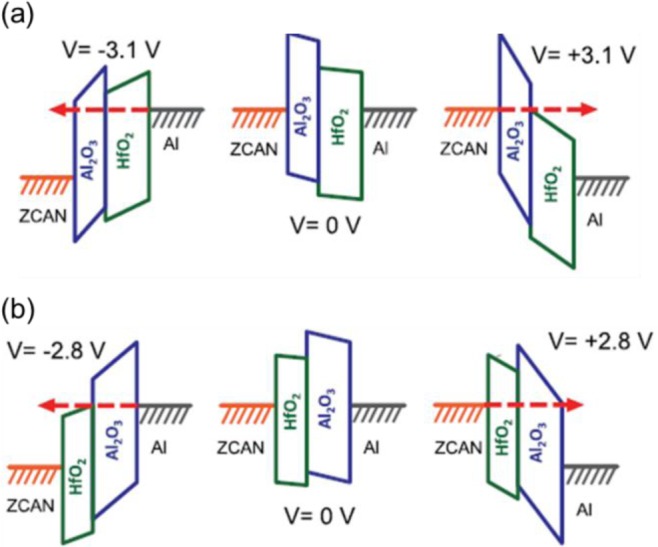
Band diagrams illustrating metal-insulator-insulator-metal (MIIM) diodes under negative bias (left), neutral (center) and positive bias (right) for diodes designed for higher current flow under (**a**) positive bias and (**b**) negative bias. Reprinted with permission from [156]. Copyright 2013 American Institute of Physics.

MIM Diodes have recently been fabricated using HfO_2_ as the insulator which bear a striking resemblance to the MIM structures used in ReRAM [154,155]. By using electrodes with asymmetric work functions, e.g., one high and one low work function, current rectifying behavior can be realized. Interestingly, one problem with the crosspoint memory arrays proposed for ReRAM devices is the phenomenon of sneak currents flowing backwards through a device adjacent to the device being read in the crosspoint array. This problem is general to crosspoint memory arrays and not just in ReRAM devices. Incorporating a diode in series with the crosspoint memory device to act as a select device is one potential solution to this problem. The use of simple MIM diodes with only HfO_2_ as the dielectric is unlikely because they do not show highly non-linear current flow. However, it was recently demonstrated that by incorporating an additional High K dielectric, such as Al_2_O_3_, the diode behavior could be enhanced [156]. The mechanism for enhanced current rectification is illustrated by the band diagrams shown in Figure 11 for forward and backward biasing. Interestingly, the dielectric stacks employed are the same as those proposed for ReRAM as described above, which suggests that it could be possible to engineer the ReRAM stack to act as its own select device- a so-called nonlinear ReRAM.

### 4.5. Ferroelectrics

Ferroelectric behavior was recently discovered in HfO_2_, ZrO_2_ and mixed HfZrO, and attributed to the non-centrosymmetric orthorhombic crystal form [157–161]. A variety of dopants has been found to select for the orthorhombic phase after crystallization [158–163]. While all of these results are quite recent, these new ferroelectric dielectrics are quite interesting as an alternative to the traditional perovskite materials that have been used in the past. These dielectrics have been incorporated into several different types of memory devices recently including ferroelectric MIM capacitors, ferroelectric floating gate memories, and ferroelectric FETs show that thinner dielectrics can be used than what was previously possible [157,163–165]. In addition a Ferroelectric PFET was found to have ultra-steep subthreshold swing (<60 mV/dec.) recently using these dielectrics [166]. All of this work has taken place just within the past three years, so there obviously remains a great deal of research and development before these dielectrics can be used for practical devices, but the ability to use simple, proven manufacturable High K dielectrics to make ferroelectric devices could open up a variety of new applications.

### 4.6. Patterning

The use of High K materials for pattern formation is another potential new application. High K metal oxides, such as Al_2_O_3_and HfO_2_, require different etch chemistries than SiO_2_, Si, or Si nitrides to remove them- meaning selective etches are available to etch any of these materials if a High K dielectric is used as the mask layer [16,167–169]. Area selective ALD has been used to form patterns with HfO_2_ serving as the hardmask [16] and Al_2_O_3_ has shown promise as a hardmask layer in various studies [170–173]. Considering that ALD Al_2_O_3_ can be deposited by ALD at temperatures well below 100 °C and even approaching room temperature on polymer substrates [174,175], it is potentially useful even for deposition on photoresist materials, and certainly for deposition on a variety of substrates at temperatures below 400 °C, the typical thermal budget allowed for back end of line processing. Other High K dielectrics have also been deposited at extremely low temperatures but the characteristics of the TMA based ALD Al_2_O_3_ make it perhaps the most ideal system for ALD film deposition [31,176–178]. Thus far, this area does not seem to have been well explored, but it is unlikely to be ignored as the ability to deposit High K films has been added to the Integration Engineers’ toolbox.

## 5. Conclusions

The current status of High K dielectrics in VLSI manufacturing for leading edge DRAM and CMOS applications was summarized along with the deposition methods and general equipment types employed. Emerging applications for High K dielectrics in future CMOS were described, including devices employing Higher K dielectrics, Gate All Around architectures, and high mobility channels. Additional emerging applications for High K dielectrics include Resistive RAM memories, MIM diodes, Ferroelectric logic and memory devices, and as mask layers for patterning. ALD is a likely and proven deposition method for all of the applications discussed for use in future VLSI manufacturing. Each of the applications discussed is promising for use in 10 nm and beyond nodes.

## Figures and Tables

**Table 1.** Comparison of State of the Art High Performance Fully Depleted Devices Reported for the 22 nm and Below Nodes at *V*_dd_ = 0.75 V and 100 nA/μm Ioff from [22–25].

Technology and Node	CGP * (nm)	Fin Pitch (nm)	L (nm)	N/P DIBL (mV/V)	N/P SS (mV/dec)	N/P Ion (mA/μm) By Weff **	N/P Ion (mA/μm) By Device Footprint	N/P Ion (nA) per Fin
Intel 22 nm Tri-Gate	90	60	30	~50	71/72	0.88/0.74 ^#^	1.08/0.91	65/55
TSMC 16 nm FinFET	64	48	30	52/42	73/71	–	0.94/0.98 ^##^	45/47
STMicro 14 nm FDSOI	100	N/A	20	73/85	90/97	0.86/0.82	0.86/0.82	–

* Contacted Gate Pitch (CGP);

# See Table 1 in [24];

** Effective device width (Weff) is the linear channel width;

## Estimated based on reported Ion/Ioff.
